# THE RAFT DEBATE: GETTING UNDERGRADUATE STUDENTS TO THINK CRITICALLY ABOUT AGING

**DOI:** 10.1093/geroni/igae098.3116

**Published:** 2024-12-31

**Authors:** Andrew Fiscella

**Affiliations:** University of South Florida, Tampa, Florida, United States

## Abstract

A student-centered approach has been advocated to help students develop as learners, as it allows students to think critically, beyond what might be required in an essay. However, it can be difficult to achieve in larger classes, where a teacher-centered approach is more often utilized. The current study explores adapting a traditional debate format for students in a Physical Changes & Aging course as a tool to engage them in critical analysis of various topics related to biological aging, allowing the incorporation of student-centered techniques. In the Raft Debate, students represent gerontologists who become stranded on a desert island. There is a raft which can take them off the island, but it is only big enough for one group. To determine who gets off the island, students are split into teams, with pairs of teams assigned a topic which had been briefly introduced over the course of the semester. Students will be asked to prepare not only arguments in favor of their position but must also effectively counter-argue their opponents. After arguments by both teams have been presented, the audience will vote for the team which made the most convincing argument. Additionally, students will complete a peer review of their teammates. The goal of this assignment is that as students collaborate with each other and ultimately, compete against their classmates, they will gain a deeper understanding of the biological aging process and how it may be applied to both their professional and personal lives.

